# The Effect of Ultrasound and Lactic Acid Fermentation on the Selected Quality Parameters and Bioactive Compounds Content in Fermented Pumpkin (*Cucurbita pepo* L.)

**DOI:** 10.3390/molecules29235586

**Published:** 2024-11-26

**Authors:** Elżbieta Radziejewska-Kubzdela, Marcin Kidoń, Angelika Kowiel, Katarzyna Waszkowiak, Krystyna Szymandera-Buszka, Marta Bednarek, Maciej Kuligowski, Joanna Kobus-Cisowska, Dominik Mierzwa

**Affiliations:** 1 Department of Food Technology of Plant Origin, Faculty of Food Science and Nutrition, Poznań University of Life Sciences, Wojska Polskiego 31, 60-624 Poznan, Poland; elzbieta.radziejewska@up.poznan.pl (E.R.-K.); aagelikaa90@gmail.com (A.K.); marta.bednarek@up.poznan.pl (M.B.); maciej.kuligowski@up.poznan.pl (M.K.); 2 Department of Gastronomy Science and Functional Foods, Faculty of Food Science and Nutrition, Poznań University of Life Sciences, Wojska Polskiego 31, 60-624 Poznan, Poland; katarzyna.waszkowiak@up.poznan.pl (K.W.); krystyna.szymandera_buszka@up.poznan.pl (K.S.-B.); joannak@up.poznan.pl (J.K.-C.); 3 Division of Process Engineering, Institute of Chemical Technology and Engineering, Poznań University of Technology, ul. Berdychowo 4, 60-965 Poznan, Poland

**Keywords:** fermented vegetables, starter cultures, sonication, phenolic compounds, carotenoids, pumpkin

## Abstract

Increasing the consumption of fruit and vegetables can be achieved by creating new products. A promising method seems to be the directed fermentation of vegetables. The aim of this study was to investigate the effect of ultrasonic pretreatment (US; 25 kHz; 5 min) and the lactic acid bacteria strain (LAB; *Lactiplantibacillus plantarum* 299v and *Lacticaseibacillus rhamnosus* GG) on the quality of fermented pumpkin (*Cucurbita pepo* L.). The pumpkin was inoculated with 5 log CFU/g of specific LAB strain. Fermentation was carried out for 7 days at 35 °C. Some samples were US treated at the washing stage. During fermentation, there was an increase in the LAB count of 3 logarithmic cycles compared to the initial inoculum. For *L. rhamnosus*, preceding fermentation by US treatment contributed to an increased bacteria count of 4 logarithmic cycles. In the case of fermentation with *L. rhamnosus*, the lactic acid content was significantly higher than for *L. plantarum*. These samples are also characterized by higher sensory properties, desirability of taste, and overall desirability. Fermentation contributed to a decrease in carotenoid and phenolic compounds content and an increase in the antioxidant capacity of the pumpkins, regardless of the bacterial strain.

## 1. Introduction

Pumpkin (*Cucurbita pepo* L.) belongs to the *Cucurbitaceae* family, which includes about 130 cultivated and wild species. There are about 20 species within the genus Cucurbita [1]. From a nutritional point of view, the common pumpkin is characterized by low protein, fat and carbohydrate content, which makes it a low-calorie vegetable. The pumpkin is known and valued for its high carotenoid content [2]. The content of carotenoids in *Cucurbita pepo* ranges from 242 µg/100 g to 4240 µg/100 g of fresh weight [1,3]. This raw material is also a phenolic compound source, including phenolic acids and flavonols [1]. Studies show that pumpkin consumption plays an important role in the prevention of cancer, as well as exhibiting antioxidant, anti-diabetic, anti-inflammatory, and immunomodulatory properties [4].

Pumpkins are mostly processed into soups, purees, or constitute ingredients in other products, for example, jam or frozen vegetable mixtures. Fermentation is not a popular method applied to pumpkins. However, it could be an alternative way to process this raw material.

Lactic acid bacteria (LAB) have a safe metabolic activity when growing in plant materials. They convert available sugars into organic acids and other metabolites that lead to preservation and improved food quality. Fermentation also modifies and improves the sensory properties of food [5]. Another benefit of the fermentation process is that it can increase the antioxidant activity of a product [6,7,8,9]. Many authors analyzing the antioxidant activity of plant materials before and after fermentation show that fermented samples have higher antioxidant activity. This improvement can be caused by increasing the bioactive compounds content, releasing bounded phenolics from cell walls or enhancing enzymatic activity [10,11]. The process of food fermentation with probiotic LAB leads to a lower pH value, which creates unfavorable conditions for the growth of pathogenic microorganisms [12]. Lactic fermentation is most often spontaneous; however, the use of fermentation with starter cultures is preferable because it allows for greater reproducibility and standardization of products [13,14].

There is a growing interest in using vegetables as substrates for LAB growth as they offer promising opportunities to create products with bioactive potential [15]. Mainly bacteria in the order *Lactobacillales* are able to synthesis lactic acid from sugars. *Lactiplantibacillus plantarum* is one of the most diverse and adaptive species of LAB. It is a bacteria strain capable of adapting to various environments, including dairy products, sourdoughs, fruits, vegetables, cereals, meat, fish, and the mammalian gastrointestinal tract [13]. A study by Liu et al. [16] showed that fermentation of sea buckthorn using *L. plantarum* yielded a product with favorable sensory characteristics. On the other hand, *Lacticaseibacillus rhamnosus* GG exhibits strong antimicrobial properties [17]. Studies have shown that it has the ability to produce ferulic acid (known for its antioxidant and antimicrobial properties) in various concentrations, enhancing its health-promoting and protective potential. *L. rhamnosus* is crucial in improving the physicochemical properties of fermented plant products such as soybeans and hazelnuts [18,19]. Studies conducted by Muncey and Hekma [20] have shown that *L. rhamnosus* significantly reduces the fermentation time of these raw materials.

In recent years, there has been growing interest in ultrasound waves in food processing, including fermentation processes. According to Chen [21], sonication can increase the availability of nutrients by disrupting cell membranes and promoting their release into the environment. It may contribute to better microbial growth on the plant matrix. Ultrasound waves can be used both at the fermentation stage and before the process during pretreatment. Studies show improvement in microorganism enzymatic and metabolic activity during fermentation [19,22]. The use of sonication at the pre-fermentation stage has not been investigated so far.

The aim of the present study was to determine the effect of pumpkin fermentation with *Lactiplantibacillus plantarum* 299v and *Lacticaseibacillus rhamnosus* GG strains on the content of bioactive compounds, physicochemical and sensory properties of the fermented product. It was also focused on the impact of the ultrasonic treatment applied before fermentation on this quality.

## 2. Results and Discussion

### 2.1. LAB Count in the Raw and Fermented Pumpkin

**Figure 1:**
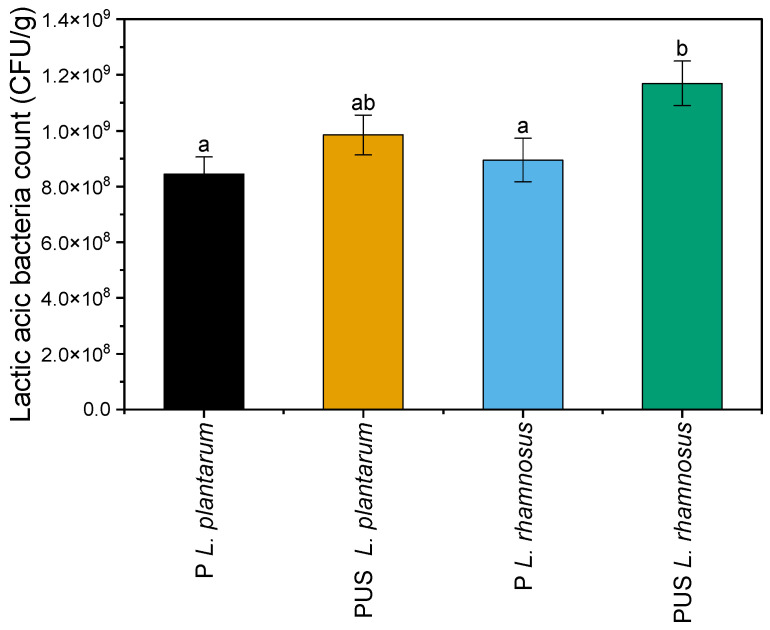
LAB count (CFU/g) in the raw and fermented pumpkin samples. a, b—the means indicated by the different letters above the columns differ significantly at the α ≤ 0.05 significance level; P *L. plantarum*—pumpkin fermented with *L. plantarum*; PUS *L. plantarum*—pumpkin treated with US and fermented with *L. plantarum*; P *L. rhamnosus*—pumpkin fermented with *L. rhamnosus*; PUS *L. rhamnosus*—pumpkin treated with US and fermented with *L. rhamnosus*.

After 7 days of fermentation, the increase in the bacteria count was observed by three logarithmic cycles compared to the initial inoculum, irrespective of the strain used. When the fermentation with the *L. rhamnosus* strain was preceded by ultrasonic treatment, an increase in LAB count was recorded by four logarithmic cycles (Figure 1). Liu et al. [16] indicate that the ultrasound application may lead to a cell membrane damage and nutrient release, contributing to the intensification of LAB growth.

### 2.2. pH Value and Soluble Solid Content of the Raw and Fermented Pumpkin

**Figure 2:**
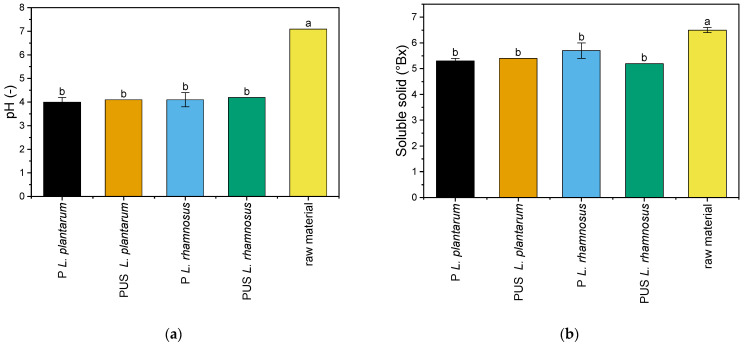
Physicochemical properties of the raw and fermented pumpkin samples: (**a**) pH value; (**b**) soluble solid content. a, b—the means indicated by the different letters above the columns differ significantly at the α ≤ 0.05 significance level; P *L. plantarum*—pumpkin fermented with *L. plantarum*; PUS *L. plantarum*—pumpkin treated with US and fermented with *L. plantarum*; P *L. rhamnosus*—pumpkin fermented with *L. rhamnosus*; PUS *L. rhamnosus*—pumpkin treated with US and fermented with *L. rhamnosus*.

The pH value and soluble solid content for the unfermented (raw) pumpkin samples were 7.1 and 6.5° Bx, respectively (Figure 2). Biesiada et al. [3] and Nawirska-Olszańska et al. [23] studying various pumpkin cultivars found that the pH value ranged from 5.99–7.10 and the soluble solid content from 2.9 to 7.4° Bx. Thus, both values for the tested raw material in this study were rather in the upper limits of the ranges determined.

Fermentation resulted in a significant decrease in pH value and soluble solid content of the fermented pumpkin samples. After 120 h of pumpkin fermentation, the pH value was in the range of 4.0–4.2. There is no data on pumpkin fermentation in the literature. Kohajdová et al. [24] recorded a pH value of 3.6 for 72 h fermented pumpkin juice with *L. plantarum* strain. Dimitrovski et al. [25] achieved a pH value of 3.6 after 33 h fermentation with *L. casei*. The fermentation process efficiency could influence such parameters as substrate availability, inoculum size, method of inoculum introduction, or process temperature [15]. In our experiment, there was no effect of the ultrasound preceding the fermentation or the LAB bacteria strain on the values of these parameters.

### 2.3. Sugar and Acid Content of the Raw and Fermented Pumpkin

**Figure 3:**
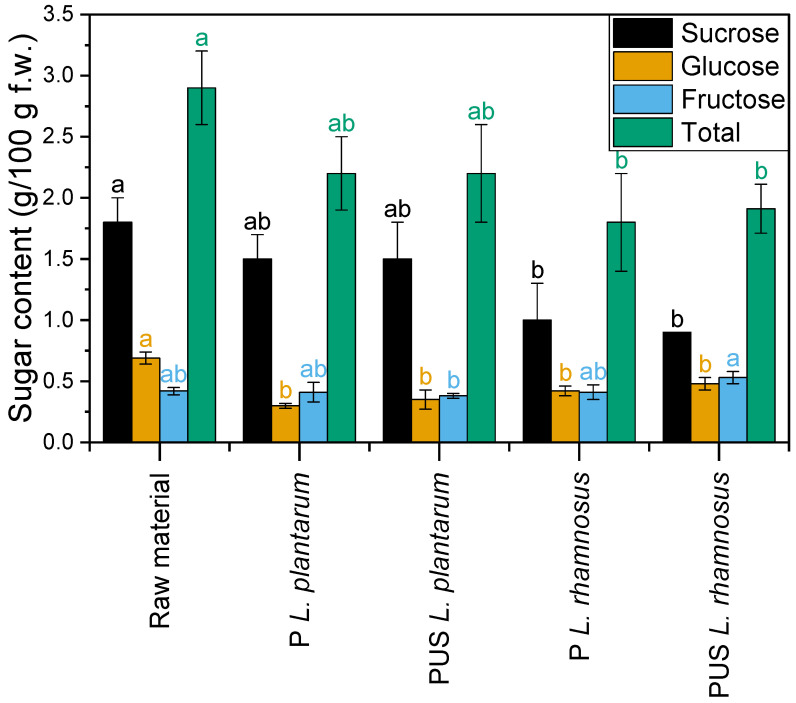
Sugar content (g/100 g f.w.) of the raw and fermented pumpkin samples. a, b—different letters above the columns of the same color indicate statistically significant differences at α ≤ 0.05 as determined by Tukey means comparison; P *L. plantarum*—pumpkin fermented with *L. plantarum*; PUS *L. plantarum*—pumpkin treated with US and fermented with *L. plantarum*; P *L. rhamnosum*—pumpkin fermented with *L. rhamnosus*; PUS *L. rhamnosum*—pumpkin treated with US and fermented with *L. rhamnosus*.

In the case of the raw pumpkin samples, the total sugar content was 2.9 g/100 g (Figure 3). Sucrose, glucose, and fructose were identified in the sugar profile. The sucrose content was predominant (1.8 g/100 g). Nawirska-Olszańska et al. [23] investigated the sugar content of several pumpkin cultivars and have shown the total sugar content in the range of 2.34–3.83 g/100 g. However, reducing sugars were predominant (1.78–2.77 g/100 g) in those profiles. The study by Zinash and Woldetsadik [26] indicates that the sugar profile may depend on the plant growing conditions.

In the tested samples, fermentation with LAB bacteria strains caused a decrease in glucose content. This decrease was about 50% and 35% for *L. plantarum* and *L. rhamnosus*, respectively. The fructose content was at the level of its content in the raw samples. However, there was a significant decrease in the sucrose content in the fermented samples with *L. rhamnosus*. The highest decrease in the sucrose content in the sample with the highest number of lactic bacteria was recorded (i.e., PUS *L. rhamnosus*).

Corcoran et al. [27] indicate that *L. rhamnosus* cannot metabolize sucrose in an acidic environment. However, the study by Bernat et al. [19] shows that sucrose content decreased after the fermentation of hazelnut milk with *Lactobacillus rhamnosus* GG. Hedberg et al. [28] also found sucrose fermentation ability for the *L. rhamnosus GG* strain, while no activity was found in *L. rhamnosus* LB21. The observations may indicate the influence of environmental pH and strain on sucrose fermentability. Bonestroo et al. [29] found that strains showing high constitutive levels of 3-fructofuranosidase and sucrose hydrolase can ferment sucrose. The ability of lactobacilli bacteria to degrade sucrose may contribute to the increase in glucose and fructose content. Thus, the relatively small decreases in the sugars in the tested samples may be related to the simultaneous hydrolysis of sucrose [30].

The total acid content in the raw pumpkin samples was 4.6 g/kg. Citric and malic acid were identified (Table 1). The first was predominant. Usenik et al. [31] and Veberic et al. [32] also found that pumpkin is a good source of citric and malic acid compared to vegetables and fruits. Nawirska-Olszańska et al. [23] additionally identified fumaric acid in the organic acid profiles of various pumpkin cultivars, but this acid was not identified in our study.

Pumpkin fermentation significantly decreased citric and malic acid content, independent of the ultrasonic pretreatment and the bacterial strain. In the case of the pumpkin fermented with *L. rhamnosus*, the content of the produced lactic acid was significantly higher than for *L. plantarum*. Moreover, a higher sucrose consumption was in the samples. It can be assumed sugars (including glucose and sucrose) and organic acids (citric and malic acid) were involved in lactic acid production.

Di Cagno et al. [33] and Caballero-Guerrero et al. [34] have found that the use of unconventional carbon sources in fermentation may be the result of microorganisms adapting to stress conditions, such as high acidity or the presence of phenolic compounds that inhibit their growth. The literature data suggest that *L. plantarum* and *L. rhamnosus* can utilize citric or malic acid as a carbon source, whereby citric acid fermentation is often accompanied by glucose utilization [35,36]. This is consistent with the results obtained in our study. The metabolic pathway for the citric acid conversion to lactic acid involves citrate lyase, an enzyme that can convert citrate to oxaloacetate. Subsequently, pyruvate can be formed by decarboxylation and converted to α-acetolactate [37]. The literature indicates that most lactic bacteria can metabolize malic acid to lactic acid. The process involves the malolactic enzyme via decarboxylation [38].

### 2.4. Color Parameters

Color parameters L*, a*, and b* of raw pumpkin flesh were 48.0, 24.4, and 52.0, respectively (Table 2). Fermentation with LAB strains caused slight changes in the color parameters of the fermented samples. Lightness increased by about 5%, and redness (a*) increased by about 13%. In predominant yellowness (b*), the highest increase was about 25%. The direction and intensity of color changes were not dependent on the LAB strain used for fermentation. Ultrasound treatment before fermentation also did not influence color parameters. The fermentation process generally caused increased saturation of color and vivid characteristics.

Itle and Kabelka [39] investigated the color of different varieties of common pumpkin flesh. They found that L* was in the range of 76.5–83.8, parameter a* was from −2.2 to 9.4 and parameter b* was from 9.8 to 64.5. The observed color of pumpkin flesh was mainly light yellow and yellow-orange. In turn, Xu et al. [40] studied the peels of light green, yellow, and orange pumpkin and for the latter, recorded color parameters similar to the raw material studied. The raw and fermented pumpkin samples investigated in our study were darker (lower L* values) and strongly reddish (higher a* values). The visual color of the pumpkin after fermentation was more orange. The differences in the values of the color parameters may also influence the fact that the measurement was in the sample homogenized with the peel. Gonçalves et al. [41] also analyzed color of the raw and blanched pumpkins; they found the following mean color values for unblanched pumpkins: L* = 59.48, a* = 26.58, and b* = 54.45, which was very similar to our results.

### 2.5. Sensory Quality of the Raw and Fermented Pumpkin

The interest in food products in the market is related to their sensory quality, especially consumer desirability [42]. The results of consumer analysis confirmed that the samples of all tested variants contained similar aroma and color desirability (Table 3). The products were rated 7.46–8.50 points for color and 5.00–9.80 points for aroma on a 10-point scale (Table 3). The differences in consumer acceptance were only for the sample taste and overall desirability. A statistically significant higher sensory desirability of taste and overall desirability was for the fermented pumpkin samples P *L. rhamnosus* and PUS *L. rhamnosus*.

The statistical analysis revealed a significant relationship between the sample’s overall desirability and the taste descriptors (r = 0.99). Therefore, they were selected for further data interpretation.

During the sensory profiling of fermented pumpkins, the following taste descriptors were defined and determined: sour, sweet, bitter, metallic, salty, silage, mouldy, tart, lactic acid, typical pumpkin, and comparable to the unfermented product (Table 4).

Analysis of the sensory profile of taste confirmed the high intensity of sour (3.98–5.40), silage (3.83–5.83), and bitter (0.57–2.38) taste in the tested samples. The highest sweet taste intensity was for P *L. rhamnosus* samples (1.57). PUS *L. plantarum* and P *L. plantarum* samples had a higher intensity of tart taste than P *L. rhamnosus* and PUS *L. rhamnosus*. The highest intensity of bitter taste (2.38) and silage taste (5.83) was also for PUS *L. plantarum* samples.

Among the selected taste descriptors, significant positive correlations were recorded between overall desirability or taste desirability and sour taste or taste comparable to unfermented raw material (Table 5). In contrast, a significant negative correlation was for tart, bitter, and lactic acid taste. The earlier studies also confirm lower acceptance of vegetable among people for bitter taste [43].

### 2.6. Carotenoid Content of the Raw and Fermented Pumpkin

Pumpkin varieties are good sources of carotenoids. Seven different carotenoids were detected in pumpkin flesh (Table 6). The main compound in the raw pumpkin samples (i.e., before fermentation) was β-carotene and its share was 75% of total carotenoids. Moreover, the raw pumpkin samples also contained considerable amounts of lutein. The total carotenoid content in the raw pumpkin samples was 60 µg/100 g.

Martinez-Valdivieso et al. [44] studied 22 pumpkin cultivars and found that the carotenoid content for exocarp ranged from 480 to 37,000 µg/100 g f.w., while for mesocarp, it ranged from 75 to 1078 µg/100 g f.w. Kulczyński and Gramza-Michałowska [1] determined the carotenoid content in 9 cultivars of *Cucurbita pepo* at levels ranging from 242 to 4240 µg/100 g f.w. with lutein being dominant in the profile. In contrast, Kim et al. [45] determined that β-carotene and β-cryptoxanthin were dominant in the pumpkin carotenoid profile. Cvetković et al. [46] found that β-carotene was dominant in the exocarp. Thus, the dominant contribution of this compound to the profile may be due to the use of raw material with peel for fermentation. The literature indicates that zeaxanthin and lycopene were also determined in the carotenoids profile of pumpkin [44,45]. The presence of neoxanthin, violaxanthin, and antheraxanthin was indicated first by Martinez-Valdivieso et al. [44]. However, they used the peel of the raw material for their study, which may indicate that these compounds are mainly found in the exocarp. The varying profiles and contents of the compounds depends on factors such as growing conditions, climate, degree of maturity, and variety [47].

After fermentation, the carotenoid content decreased significantly and achieved a content from 29 to 45 µg/100 g. A significant decrease in the β-carotene content was in the fermented samples. Its content was about 12–17 µg/100 g, i.e., only about 32% of the initial content. A significant decrease in neoxanthin and violaxanthin content was in the PUS *L. plantarum* samples. Despite that, the lutein content increased by about 10 µg/100 g after fermentation, especially in the PUS *L. plantarum* and P *L. rhamnosus* samples. An increase in content was also recorded for cryptoxanthin and in the most fermented samples for antheraxanthin.

Muntean [48], investigating the stability of carotenoids during spontaneous lactic fermentation of *Cucurbita pepo* L. var. giromontina, also found a decrease in their content. Violaxanthin was the least stable, followed by neoxanthin and lutein; β-carotene was the most stable. However, the literature shows that the effect of fermentation on carotenoid content can vary. For instance, Bartkiene et al. [49] investigating the effect of fermentation using different strains (*Lactobacillus sakei* KTU05-6, *Pediococcus acidilactici* KTU05-7, and *Pediococcus pentosaceus* KTU05-8) on the stability of β-carotene in tomato found a significant effect of variety. In the Cunero cultivar, they recorded a 25% decrease in the content of this compound, while in the Ronaldo cultivar there was an approximately 70% increase. Sangija et al. [50] point to the possibility of an increase in carotenoid content during fermentation through the enzymatic activity of lactic bacteria which explains its better extractability. On the other hand, disruption of the cellular structure due to fermentation may also lead to degradation of carotenoids through enzymatic oxidation. Changes in individual carotenoid compounds occurred in different ways. Therefore, these effects require further research.

### 2.7. Phenolic Compound Content of the Raw and Fermented Pumpkin

The content of phenolic compounds in the raw material was 7.6 mg/100 g f.w. (Table 7). Kulczyński et al. [51], studying 13 pumpkin cultivars, determined the content of phenolic compounds in a similar range of 3.09–15.17 mg/100 g. Cinnamic, syringic, and p-coumaric acids predominated in the phenolic acid profile. Gallic, hydroxybenzoic, protocatechuic, vanillic, and ferulic acids also were identified. According to the literature, benzoic acid, sinapic acid hexoside, 2,4,5 tri-o galloylquinic acid, chlorogenic acid, rutin, luteolin, quercetin, and isoramnetin were also identified in addition to the mentioned compounds [1,52,53]. The study by Stryjecka et al. [54] and Kulczyński and Gramza-Michałowska [1] show that the content of individual phenolic compounds is determined the most by cultivar. Research also suggests that these compounds play an important role in the prevention of many diseases. For instance, syringic and coumaric acids may present anti-diabetic properties by inhibition of α-amylase and α-glucosidase. Procatechuic and hydrobenzoic acids are associated with antimicrobial, anti-inflammatory, antihyperglycemic, antiapoptotic, and antiproliferative effects [55].

Fermentation contributed to a decrease in phenolic content of around 30%, irrespective of the bacterial strain used. Preceding the fermentation process with ultrasonic treatment increased the loss of the compounds by a further 30–40%. Decreases were in the content of cinnamic, p-coumaric, syringic, gallic, protocatechuic, and vanillic acids. Moreover, the ultrasound effect on the significant reduction in cinnamic, vanillic, syringic, and *p*-coumaric acid contents was in the case of pumpkin samples fermented with *L. plantarum*. In contrast, the contents of hydroxybenzoic and ferulic acids in the fermented and ultrasonic pre-treated samples were at the level of their content in the raw material.

A 22–34.8% decrease in phenolic compounds after the fermentation of apple juice with the *L. plantarum* strain was found by Li et al. [56]. Zhang et al. [57] also reported a reduction in these compounds’ content in *Caucasian hurma* after fermentation. However, there is also literature data indicating a significant increase in phenolic acids in fermented vegetables or fruits [58]. Changes in the content of phenolic compounds can be influenced, among other things, by bacterial enzyme activity such as esterases, decarboxylases, and reductases [59]. The literature suggests that the metabolism of phenolic compounds by lactic fermentation bacteria is also highly dependent on the plant matrix. Filannino et al. [60] found an approximately 70% decrease in protocatechuic acid after the fermentation of cherry juice with strains of *Lactoplantibacillus plantarum* and its conversion to catechol. They also noted the consumption of *p*-coumaric acid by *L. plantarum* strains and accumulation of phloretic acid. However, these transformations are also dependent on the bacterial strain, substrate availability in the plant matrix, fermentation temperature, or time [59]. Therefore, determining their course would require model studies.

The reduction in phenolic compounds may also be related to their leaching into the fermentation broth. Additionally, acoustic waves can induce unstable cavitation in solution, which results in a plant tissue membrane disruption and higher phenolic compound extractability [61].

### 2.8. Antioxidant Activity of the Raw and Fermented Pumpkin

**Figure 4:**
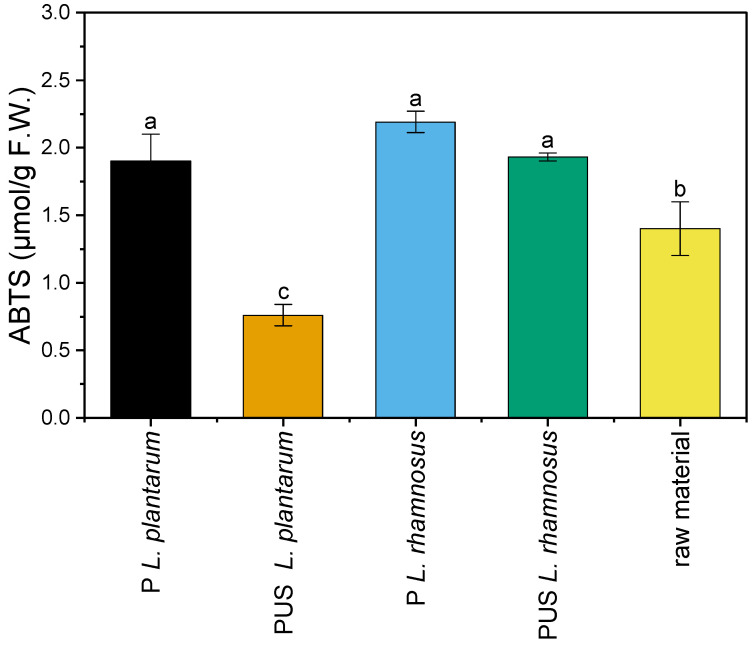
Antioxidant activity of the raw and fermented pumpkin samples. a, b, c—the means indicated by the different letters above the columns differ significantly at the α ≤ 0.05 significance level; P *L. plantarum*—pumpkin fermented with *L. plantarum*; PUS *L. plantarum*—pumpkin treated with US and fermented with *L. plantarum*; P *L. rhamnosus*—pumpkin fermented with *L. rhamnosus*; PUS *L. rhamnosus*—pumpkin treated with US and fermented with *L. rhamnosus*.

The determined antioxidant activity for the raw material was 1.4 µmol/g (Figure 4). Antioxidant activities of 13 pumpkin cultivars were determined at a similar level (0.70–4.63 µmol/g) by Kulczyński et al. [51]. The fermentation of the pumpkins increased the fermented sample’s antioxidant capacity, irrespective of the applied strain. A statistically significant decrease in antioxidant activity was observed only for the PUS *L. plantarum* sample.

Zhou et al. [62] and Hashemi and Jafarpour [63] also reported the increased antioxidant activity of kiwi juice and bergamot juice after fermentation, irrespectively. They related it to phenolic content increase, their release from bound forms, or their transformation. In the case of pumpkin fermentation in our research, there was no significant correlation between phenolic compound content and antioxidant activity.

Many studies suggest that phenolic compounds are responsible for antioxidant activity. However, it should be mentioned that antioxidant activity possesses large and diversified groups of molecules, with different chemical structures and activities (e.g., vitamins, other plant metabolites, etc.) [64]. The increase in the antioxidant capacity for most fermented samples may be related to the production of exopolysaccharides by lactic fermentation bacteria. The study of Lyu et al. [65] reported such a relationship. Regardless of the type of compounds responsible for that, high antioxidant activity of foods is desirable. Oxidative reactions could lead to deterioration of food and human health. Foods with high antioxidant activity may play an important role in preventing oxidative degradation and pathological processes [66].

## 3. Materials and Methods

### 3.1. Pumpkin Fermentation

The pumpkin (*Cucurbita pepo* L.) was purchased during the 2023 season from a local farmer. The pumpkin was cut into quarters with a kitchen knife. The soft flesh with seeds was then removed and the quarters were cut in half. The pumpkin pieces were then washed using an industrial vegetable brush washer. The washer was equipped with a 2 × 4 kW ultrasonic generator with a frequency of 25 kHz. Half of the prepared pumpkin pieces were subjected to ultrasound for 5 min during washing. The washed vegetables were then fermented. Portions of approximately 10 kg of pumpkin pieces were placed in buckets lined with plastic bags and poured into a brine consisting of a 3% salt–water solution. The ratio of vegetables to brine was approximately 1:1. Commercially available LAB probiotic strains *L. plantarum* 299v and *L. rhamnosus* GG were used for fermentation. The freeze-dried microorganisms were dissolved in a 3% brine and added to buckets of vegetables in brine. The concentration of microorganisms was 5 log CFU per g of vegetables. The pumpkin pieces were fermented for seven days at 35 °C in sealed tightly in bags. After this time, they were separated through a sieve from the brine and homogenized into a puree. Samples for microbiological analysis were taken immediately after fermentation. The remainder of the fermented puree was transferred to glass jars of approximately 0.9 L and pasteurized for 20 min at 90 °C. Then, the fermented puree was cooled to room temperature and subjected to other analyses.

### 3.2. Determination of LAB Counts [67]

About 10 g of pumpkin sample was mixed with 90 mL of sterile peptone solution and shaken for 30 min. Ten-fold dilutions were prepared from these samples. LAB was determined using a MRS (De Man–Rogosa–Sharpe) agar (Merck, Darmstadt, Germany) and the pour plating method after incubation at 37 °C for 72 h. Then, the colonies were counted.

### 3.3. Determination of pH and Soluble Solids

Determination of pH was conducted using an electronic pH-meter HI 2223 (Hanna-Instruments, Smithfield, VA, USA). The total soluble solids were determined using a digital refractometer HI 96801 (Hanna Instruments, Woonsocket, RI, USA) at 20 °C.

### 3.4. Determination of Sugars Content

In the raw and fermented samples, sugar contents were analyzed according to the Kelebek method [68]. Every sample (10 g) was homogenized with 10 mL of distilled water and extracted for 10 min in a mechanical shaker. Then, samples were centrifuged at 5200 rpm for 15 min. Supernatant was collected, and the remaining residue was reextracted with water. The supernatants were combined and filled to 25 mL with distilled water. Before analysis, the sample was filtered through a 0.45 µm pore-size membrane filter. An Agilent 1260 Infinity HPLC system (Agilent Technologies, Palo Alto, CA, USA) equipped with Shodex Sugar EP-SC1011-7F 8 µm, 7.8 × 300 mm (Resonac America Inc., New York, NY, USA) column and a refractive index detector (RID 1260 Infinity II, Agilent Technologies, Palo Alto, CA, USA) was used for sugar analysis. An eluent water was used with the flow rate of 0.5 mL/min at 70 °C.

### 3.5. Determination of Organic Acid Content

Organic acid content was determined according to the Kelebek procedure [68] using the same extraction method and HPLC system as for sugar analysis. Organic acid was separated at a Rezex ROA-Organic Acid H+ 8 µm, 7.8 × 300 mm (Phenomenex, Torrance, CA, USA) column at 55 °C, with 0.09 M H_2_SO_4_ in 6% acetonitrile (*v*/*v*) at flow rate 0.5 mL/min. The organic acid detection was conducted by a DAD detector at wavelength 210 nm.

### 3.6. Analysis of Color

The color parameters of the CIELAB color system were measured using the Konica Minolta 3600d spectrophotometer (Konica-Minolta, Osaka, Japan). The samples were transferred into a glass cuvette with 25 mm thickness and placed into the spectrophotometer. Measurement in reflectance mode was conducted. Specular reflectance was excluded, and illuminant D65 and 2° observation angle were used.

### 3.7. Sensory Analysis

Sensory analyses were conducted in an appropriately designed and equipped sensory analysis laboratory [69] at the Department of Gastronomy Science and Functional Foods, Poznan University of Life Sciences, Poland.

The samples were coded with three-digit numbers, and the order of samples served was random. The program Analsens NT (LABNT, Warsaw, Poland) was used for coding and arrangement of serving order.

Samples (35 g) were served in biodegradable plastic containers (50 mL) covered with the lids. Unsweetened black tea (temp. ~45 °C) was used as a taste neutralizer between the samples.

Consumer analysis was conducted with 50 people aged 21–50. Women constituted 62% of the population analyzed. The criterion for inclusion into the study was consumption of fermented products (at least once a month). No ethical approval was required for this study. Participants were informed about the study’s aim and that their participation was entirely voluntary. They could stop the analysis at any point, and the responses were anonymous. The authors did not ask for sensitive data and personal information. Consumers evaluated desirability of color, taste, aroma, and overall desirability. A 10 cm unstructured linear scale was applied with appropriate margin descriptions: undesirable—highly desirable. All consumers rated all samples in one session.

The sensory profiling was conducted by a 6-member trained panel. The Sensory Profile was analyzed for taste. In the research, eleven descriptors for taste (sour, sweet, bitter, metallic, salty, silage, mouldy, tart, lactic acid, and typical pumpkin). The intensity of each descriptors score was determined using a 10 cm unstructured linear scale with appropriate margin descriptions. Uniform margin denotations were applied: “undetectable—very intensive”. All samples were assessed in two independent replications.

### 3.8. Determination of Carotenoid Content

Carotenoid contents were analyzed by de Sá and Rodriguez-Amaya methodology [70]. Sample (10 g) was homogenized with 25 mL of acetone. The mixture was shaken on a laboratory shaker for 15 min and centrifuged at 5200 rpm for 15 min. The supernatant was collected and the residue re-extracted with a new aliquot of acetone. Then, the supernatants were combined, filtered through a vacuum filter, and evaporated to dryness at 40 °C. The residue was transferred with acetone to a 10 mL volumetric flask, filtered through a 0.22 μm pore size membrane and analyzed by HPLC. An Agilent 1260 Infiniity HPLC system (Agilent Technologies, Palo Alto, CA, USA) was used, equipped with a Zorbax SBC-18 5 µm, 4.6 × 150 mm column (Agilent Technologies, Palo Alto, CA, USA). A mixture of acetonitrile and triethylamine (0.05%, *v*/*v*) and methanol and ethyl acetate (11:9, *v*/*v*) were used as solvent A and solvent B, respectively. The flow rate was 0.5 mL/min.

### 3.9. Determination of Phenolic Compound Content

Phenolic compound determination was conducted by HPLC according to Tsao and Yang [71]. A sample (10 g) was weighed and suspended in 50 mL of aqueous methanol solution (70% *v*/*v*). Then, it was homogenized for 1 min, shaken for 15 min, and centrifuged at 5200 rpm for 15 min. The supernatant was collected, and the remaining residue was re-extracted with a new portion of 70% methanol. Supernatants were combined, filtered through a vacuum filter, and evaporated at 45 °C. The residue was transferred to a 25 mL volumetric flask and filled with distilled water. Then, the sample was filtered by a 0.45 μm pore size membrane and subjected to HPLC analysis. An Agilent 1260 Infinityy HPLC system (Agilent Technologies, Palo Alto, CA, USA), equipped with Zorbax SBC-18 5 µm, 4.6 × 150 mm column (Agilent Technologies, Palo Alto, CA, USA) was used for the analysis. A solution of 6% acetic acid in 2 mM sodium acetate and pure acetonitrile was used as solvent A and B, respectively. The flow rate was 1 mL/min.

### 3.10. Antioxidant Activity

The antioxidant activity was determined using ABTS method of Re et al. [72]. An ABTS radical was generated chemically by mixing in equal volumes of 7.4 mM ABTS+ solution and 2.6 mM potassium persulfate solution. The mixture was left in the dark for 12 h at room temperature. Then, ABTS radical was diluted with PBS 7.4 pH buffer to obtain absorbance 0.70 ± 0.02 at 734 nm. The appropriate dilution of phenolic extracts was added and mixed. The mixture was allowed to react for 6 min at 30 °C. Then, the absorbance was read at 734 nm using the spectrophotometer. Results were expressed as μM Trolox equivalent per gram fresh weight.

### 3.11. Statistical Analysis

All determinations were carried out in triplicate. Statistical analysis was performed using Statistica 12 software (TIBCO Software Inc., Palo Alto, CA, USA). Data were subjected to analysis of variance (ANOVA) and means were compared using the post-hoc Tukey test at the significance level. Relationships between variables were assessed using Pearson’s correlation coefficient.

## 4. Conclusions

The effect of ultrasound pretreatment and further fermentation with LAB bacteria (i.e., *L. plantarum* and *L. rhamnosus*) on pumpkin quality was investigated for the first time in this study. The bacterial strain used did not affect bacterial growth during pumpkin fermentation. The ultrasound applied at the pretreatment stage resulted in significantly higher bacterial growth (by one logarithmic cycle) only for the *L. rhamnosus* strain. The main carbon sources during fermentation were glucose and citric acid, regardless of the strain used. Moreover, sucrose was metabolized during pumpkin fermentation with *L. rhamnosus*; simultaneously, a significantly higher lactic acid content was noted in the samples. The samples fermented with *L. rhamnosus* were also characterized by higher sensory acceptability. The poorer flavor evaluation of the fermented samples was influenced by tart, bitter, and lactic acid taste intensity. In contrast, the sour taste and the taste comparable to unfermented raw material was desirable. Determining the descriptors responsible for the desired taste of fermented pumpkin can be helpful in the quality development of product with fermented pumpkin.

Pumpkin fermentation resulted in a significant decrease in phenolic compounds and carotenoids, irrespective of the LAB strain used. At the same time, the ultrasound at the pretreatment stage increased the loss of phenolic compounds. However, an increase in antioxidant activity was found after fermentation, but it did not correlate with the content of phenolic compounds in our study. Thus, this aspect requires further research.

Among the samples tested in the study, pumpkin fermented with the *L. rhamnosus* strain was of better quality than *L. plantarum*. The application of ultrasound did not improve the quality of the pumpkin. The semi-product can be used as a component, for example, fermented vegetable spreads, which can expand the range of nutritious fermented vegetable products.

## Figures and Tables

**Table 1:** Acid content (g/kg f.w.) of the raw and fermented pumpkin samples.

Sample	Citric Acid	Malic Acid	Lactic Acid	Total
Raw material	2.9 ± 0.8 a	1.6 ± 0.2	nd	4.5 ± 0.9 a
P *L. plantarum*	0.06 ± 0.01 b	nd	1.2 ± 0.02 b	1.8 ± 0.3 b
PUS *L. plantarum*	0.04 ± 0.01 b	nd	0.9 ± 0.00 b	1.3 ± 0.1 b
P *L rhamnosus*	0.06 ± 0.02 b	nd	2.0 ± 0.03 a	2.6 ± 0.1 b
PUS *L. rhamnosus*	0.28 ± 0.02 b	nd	2.2 ± 0.02 a	2.5 ± 0.4 b

nd—not detected; a, b—the means indicated by the different letters in the column differ significantly at the α ≤ 0.05 significance level; P *L. plantarum*—pumpkin fermented with *L. plantarum*; PUS *L. plantarum*—pumpkin treated with US and fermented with *L. plantarum*; P *L. rhamnosus*—pumpkin fermented with *L. rhamnosus*; PUS *L. rhamnosus*—pumpkin treated with US and fermented with *L. rhamnosus*.

**Table 2:** Color parameters of the raw and fermented pumpkin samples.

Sample	L*	a*	b*
Raw material	48.0 ± 1.0 c	24.4 ± 0.8 b	52.0 ± 0.8 c
P *L. plantarum*	50.4 ± 0.2 ab	27.0 ± 0.3 a	64.0 ± 2.0 ab
PUS *L. plantarum*	49.4 ± 0.2 a	27.0 ± 0.2 a	65.6 ± 0.8 a
P *L. rhamnosus*	50.6 ± 0.3 b	27.7 ± 0.2 a	62.8 ± 0.5 b
PUS *L. rhamnosus*	50.4 ± 0.3 ab	27.7 ± 0.1 a	63.2 ± 0.6 b

a, b, c—the means indicated by the different letters in the column differ significantly at the α ≤ 0.05 significance level; P *L. plantarum*—pumpkin fermented with *L. plantarum*; PUS *L. plantarum*—pumpkin treated with US and fermented with *L. plantarum*; P *L. rhamnosus*—pumpkin fermented with *L. rhamnosus*; PUS *L. rhamnosus*—pumpkin treated with US and fermented with *L. rhamnosus*.

**Table 3:** The consumer desirability of the fermented pumpkin samples. Mean scores (n = 50) ± standard deviation.

Sample	Desirability of			
	Color	Aroma	Taste	Overall
P *L. plantarum*	8.50 ± 1.15 a	6.50 ± 1.21 a	4.20 ± 1.08 b	4.02 ± 0.96 b
PUS *L. plantarum*	7.60 ± 1.26 a	6.80 ± 1.35 a	4.50 ± 1.24 b	4.61 ± 1.26 ab
P *L. rhamnosus*	8.00 ± 1.36 a	5.00 ± 1.21 a	6.56 ± 1.04 a	6.50 ± 1.16 a
PUS *L. rhamnosus*	7.46 ± 1.22 a	5.00 ± 1.11 a	6.00 ± 1.10 a	6.20 ± 1.10 a

a, b—the means indicated by the different letters in the row differ significantly at the α ≤ 0.05 significance level; P *L. plantarum*—pumpkin fermented with *L. plantarum*; PUS *L. plantarum*—pumpkin treated with US and fermented with *L. plantarum*; P *L. rhamnosus*—pumpkin fermented with *L. rhamnosus*; PUS *L. rhamnosus*—pumpkin treated with US and fermented with *L. rhamnosus*.

**Table 4:** Sensory profiling results. Mean scores (n = 6) of taste descriptors for the fermented pumpkin samples.

Taste Descriptors	P *L. plantarum*	PUS *L. plantarum*	P *L. rhamnosus*	PUS *L. rhamnosus*
Sour	3.98 ± 0.28 b	5.17 ± 0.24 a	5.40 ± 0.35 a	5.27 ± 0.25 a
Sweet	0.72 ± 0.15 c	0.68 ± 0.15 c	1.57 ± 0.08 a	1.18 ± 0.16 b
Bitter	1.35 ± 0.14 b	2.38 ± 0.16 a	0.57 ± 0.26 c	0.85 ± 0.14 c
Metallic	0.00 ± 0.00 a	0.00 ± 0.00 a	0.00 ± 0.00 a	0.00 ± 0.00 a
Salty	0.48 ± 0.19 a	0.40 ± 0.14 a	0.43 ± 0.14 a	0.47 ± 0.10 a
Silage	5.17 ± 0.28 b	5.83 ± 0.08 a	3.83 ± 0.31 c	4.08 ± 0.10 c
Mouldy	0.00 ± 0.00 a	0.00 ± 0.00 a	0.00 ± 0.00 a	0.00 ± 0.00 a
Tart	2.13 ± 0.22 a	1.75 ± 0.16 a	0.92 ± 0.20 b	0.68 ± 0.12 b
lactic acid	1.65 ± 0.16 a	1.33 ± 0.12 a	1.23 ± 0.10 a	1.02 ± 0.17 a
typical pumpkin	1.03 ± 0.10 b	1.57 ± 0.16 ab	1.88 ± 0.23 a	1.75 ± 0.31 a
compared to unfermented	1.00 ± 0.06 d	1.35 ± 0.14 c	2.53 ± 0.33 a	1.77 ± 0.19 b

a, b, c, d—the means indicated by the different letters in the row differ significantly at the α ≤ 0.05 significance level; P *L. plantarum*—pumpkin fermented with *L. plantarum*; PUS *L. plantarum*—pumpkin treated with US and fermented with *L. plantarum*; P *L. rhamnosus*—pumpkin fermented with *L. rhamnosus*; PUS *L. rhamnosus*—pumpkin treated with US and fermented with *L. rhamnosus*.

**Table 5:** The correlation coefficients between taste descriptors and taste or overall desirability of the fermented pumpkin at α ≤ 0.05.

Desirability	Sour	Sweet	Bitter	Salty	Silage	Tart	Lactic Acid	Typical Pumpkin	Compared to Unfermented
Taste	0.747	0.636	−0.787	−0.046	−0.541	−0.935	−0.768	0.398	0.872
Overall	0.809	0.546	−0.724	−0.096	−0.427	−0.971	−0.848	0.480	0.914

**Table 6:** Carotenoid content [µg/100 g f.w.] of the raw and fermented pumpkin samples.

Sample	Neoxanthin	Violaxanthin	Antheroxanthin	Lutein	Cryptoxanthin	α-Carotene	β-Carotene	Total
Raw material	0.88 ± 0.08 a	0.41 ± 0.00 ab	0.71 ± 0.06 c	12.6 ± 0.8 c	0.21 ± 0.01 d	0.80 ± 0.30 a	45 ± 2 a	60 ± 3 a
P *L. plantarum*	0.31 ± 0.07 bc	0.28 ± 0.02 bc	1.40 ± 0.20 b	18 ± 3 abc	1.35 ± 0.04 a	0.04 ± 0.01 ab	13.3 ± 0.1 bc	33 ± 4 bc
PUS *L. plantarum*	0.30 ± 0.06 bc	0.27 ± 0.01 c	2.10 ± 0.20 a	22 ± 2 a	0.50 ± 0.10 bc	0.53 ± 0.00 a	17.4 ± 0.9 b	45 ± 3 b
P *L. rhamnosus*	0.53 ± 0.10 b	0.52 ± 0.07 a	1.90 ± 0.10 a	20 ± 2 a	0.90 ± 0.01 a	0.00 ± 0.01 b	11.8 ± 0.1 c	36 ± 2 bc
PUS *L. rhamnosus*	0.11 ± 0.01 c	0.29 ± 0.02 bc	1.02 ± 0.08 cb	15.1 ± 0.8 bc	0.68 ± 0.06 b	0.36 ± 0.06 ab	12 ± 2 c	29 ± 3 c

a, b, c, d—the means indicated by the different letters in the column differ significantly at the α ≤ 0.05 significance level; P *L. plantarum*—pumpkin fermented with *L. plantarum*; PUS *L. plantarum*—pumpkin treated with US and fermented with *L. plantarum*; P *L. rhamnosus*—pumpkin fermented with *L. rhamnosus*; PUS *L. rhamnosus*—pumpkin treated with US and fermented with *L. rhamnosus*.

**Table 7:** Phenol compounds [mg/100 g f.w.] of the raw and fermented pumpkin samples.

Sample	Galic Acid	Cinnamic Acid	Hydroxybenzoic Acid	Protocatechuic Acid	Vanillic Acid	Syringic Acid	Ferulic Acid	*p*-Coumaric Acid	Total
Raw material	0.8 ± 0.1 a	2.2 ± 0.3 a	0.70 ± 0.01 a	0.30 ± 0.07 a	0.49 ± 0.09 ab	1.68 ± 0.04 a	0.19 ± 0.07 a	1.2 ± 0.1 a	7.6 ± 0.4 a
P *L. plantarum*	0.157 ± 0.001 b	1.4 ± 0.1 b	0.7 ± 0.1 a	0.12 ± 0.03 b	0.67 ± 0.03 a	1.69 ± 0.01 a	0.10 ± 0.05 a	0.46 ± 0.00 b	5.3 ± 0.2 b
PUS *L. plantarum*	0.09 ± 0.01 b	0.72 ± 0.06 c	0.4 ± 0.2 a	0.09 ± 0.01 b	0.35 ± 0.07 bc	0.85 ± 0.00 b	0.08 ± 0.01 a	0.19 ± 0.02 c	2.8 ± 0.3 c
P *L. rhamnosus*	0.094 ± 0.005 b	1.0 ± 0.2 c	0.8 ± 0.1 a	0.14 ± 0.01 ab	0.56 ± 0.07 ab	1.9 ± 0.2 a	0.06 ± 0.01 a	0.35 ± 0.04 bc	4.9 ± 0.1 b
PUS *L. rhamnosus*	0.075 ± 0.004 b	nd	0.38 ± 0.01 a	0.08 ± 0.01 b	0.22 ± 0.01 c	0.71 ± 0.06 b	0.09 ± 0.01 a	0.20 ± 0.01 c	1.8 ± 0.1 c

nd—not detected; a, b, c—the means indicated by the different letters in the column differ significantly at the α ≤ 0.05 significance level; P *L. plantarum*—pumpkin fermented with *L. plantarum*; PUS *L. plantarum*—pumpkin treated with US and fermented with *L. plantarum*; P *L. rhamnosus*—pumpkin fermented with *L. rhamnosus*; PUS *L. rhamnosus*—pumpkin treated with US and fermented with *L. rhamnosus*.

## Data Availability

The data presented in this study are available in the article.
